# Is testicular microdissection the only way to retrieve sperm for non-obstructive azoospermic men?

**DOI:** 10.3389/frph.2022.980824

**Published:** 2022-08-23

**Authors:** Marcelo Vieira, Marcos Alécio Bispo de Andrade, Eduesley Santana-Santos

**Affiliations:** ^1^Programa de Atendimento Integral ao Homem (PAIH), São Paulo, Brazil; ^2^Fellow in Andrology at Aliança de Laboratórios para Fertilização Assistida, São Paulo, Brazil; ^3^Graduate Nursing Program, Federal University of Sergipe, São Cristóvão, Sergipe, Brazil

**Keywords:** male infertility, azoospermia, sperm retrieval, reproductive techniques, intracytoplasmic sperm injection, treatment options

## Abstract

Men presenting with non-obstructive azoospermia are the most challenging clinical scenario for an infertile couple. Intracytoplasmic Sperm Injection (ICSI) with testicular sperm retrieval gave a chance for biological fatherhood once sperm can be found, but unfortunately sperm recovery rate (SSR) is something near 50%, leading to a discussion about what surgical retrieval technique is the best. Historically sperm have been retrieved using conventional Testicular Sperm Extraction (c-TESE), Testicular Sperm Aspiration (TESA), a combination of Testicular Fine Needle Aspiration (TfNA)/c-TESE, Testicular Microdissection (TM) and Open Testicular Mapping (OTEM). c-TESE published in 1995 by Devroey and cols. consists of testis delivery, a large unique albuginea incision and extraction of a portion from the majority of testicular tubules. TESA published in 1996 by Lewin and cols. is done percutaneously using a 21–23 gauge needle and a syringe to aspire testicular tubules. TfNA was published in 1965 by Obrant and Persson as an aspiration biopsy and cytological exam to verify sperm production. In 1999 Turek and cols. published the use of TfNA combined with c-TESE for sperm retrieval. In 1999, Peter Schlegel published a technique using a microsurgical approach to identify more probable sperm production areas inside the testicle that could be excised with better precision and less tissue. OTEM is a multiple biopsy approach, published in 2020 by Vieira and cols., based on TfNA principles but done at the same time without albuginea opening or surgical microscope need. Since Testicular Microdissection publication, the method became the gold standard for sperm retrieval, allowing superior SSR with minimal tissue removal, but the amount of testicular dissection to find more probable spermatogenesis areas, difficulties in comparative design studies, diversity TM results among doctors and other methods that can achieve very similar results we question TM superiority. The objective is review existing literature and discuss advantages and disadvantages of all the methods for sperm retrieval in non-obstructive azoospermia.

## Introduction

Infertility is a common clinical problem that affects 13 to 15% of couples worldwide (1). According to Kamel et al. (2), infertility should be considered a public health problem, as it affects not only the health system, but also the social environment. Azoospermia, defined as the complete absence of sperm in the semen after centrifugation in at least two samples, is observed in 1% of the general population and in 10–15% of infertile men, and can be classified as obstructive azoospermia (OA) and non-obstructive azoospermia (NOA)(3).

Non-obstructive azoospermia represents the most severe form of male factor infertility, resulting from testicular failure. This problem affects 10% of men diagnosed with infertility and is found in 60% of cases of azoospermia (4, 5). Although OA is normally characterized as presence of normal spermatogenesis, NOA represents a heterogeneous condition with impaired spermatogenesis, ranging from hypospermatogenesis and maturation arrest to Sertoli cell-only syndrome (SCO) (6–8). Although azoospermia is associated with infertility, it does not necessarily imply sterility because many azoospermic men maintain sperm production at different levels within the testes (9).

Several sperm retrieval (SR) techniques have been developed to collect sperm from the epididymis or the testicles in azoospermic men. Surgically retrieved sperm can be used to induce pregnancy through assisted reproduction techniques, i.e., *in vitro* fertilization associated with intracytoplasmic sperm injection (ICSI) (10–14).

In patients with NOA, sperm retrieval can be performed due to the existence of isolated islands of active spermatogenesis within the testicles. Different SR techniques have been described in the literature for this purpose, such as: Testicular Fine Needle Aspiration (TfNA), Conventional Testicular Sperm Extraction (c-TESE), Testicular Sperm Aspiration (TESA), Microdissection TESE (micro-TESE), and Open Testicular Mapping (OTEM) (9, 15). Until recently, c-TESE represented the first-line treatment for sperm retrieval in NOA patients for ICSI (16).

To date, there is no consensus in the literature on which SR technique has the best results in patients with NOA due to the lack of randomized clinical trials that compare the efficacy of available methods (14, 17, 18). Thus, the objective of this work is to review SR methods and their success rates, and assess the advantages and limitations of current surgical SR methods in men with NOA.

## Search strategy

A narrative review was performed where the keywords were selected from the Health Sciences Descriptors (DeCS) and the Medical Subject Headings (Mesh). The following databases were used: Cochrane Controlled Trials Register, U.S. National Library of Medicine (PubMed) and Scientific Electronic Library Online (SciELO). A gray literature search included Google Scholar, OpenThesis, and clinical trials. The Boolean operators “AND” and “OR” were used with the keywords to guide the search in the databases. The search was carried out between January and March 2022.

Original articles, reviews and meta-analyses were selected, written in English that returned from the search using the terms: male infertility; azoospermia; sperm recovery; reproductive techniques; intracytoplasmic sperm injection and treatment options. Productions not available in full were excluded, after trying to contact authors. Two reviewers (MABA and ESS) independently selected studies based on inclusion criteria. Disagreements between authors about the studies to be included were resolved by discussion among them and when necessary, a third reviewer (MV) was consulted.

## Techniques for the treatment of non-obstructive azoospermia

### Intracytoplasmic sperm injection (ICSI)

Since the advent of *in vitro* fertilization (IVF) in 1978 and ICSI in 1992, the treatment of azoospermic patients has undergone important changes, making it possible for men with infertility to generate their own offspring through one sperm per egg (10, 19). Unlike conventional IVF, where thousands of spermatozoa compete for fertilization, with a process of natural selection taking place, in ICSI the sperm is selected by the embryologist according to aspects of motility and morphology (20).

Before the advent of ICSI, the options for treatment in azoospermic patients were limited, however, this technique allowed this group of men the possibility of paternity through sperm retrieved from their own testes (12). ICSI was initially reserved for the treatment of couples with male factor infertility and was developed to improve fertilization rates with low-quality sperm and is currently used in approximately half of all IVF treatments (21). The association between IVF and ICSI and SR techniques are currently considered the gold standard for treatment of infertility in azoospermic patients, being routinely used in infertility treatment centers worldwide (22).

### Testicular fine needle aspiration (TfNA)

Percutaneous testicular aspiration can be performed on an outpatient basis under local anesthesia, which is less invasive than an open surgical procedure and is most useful for men with obstructive azoospermia (23). TfNA consists of the blind insertion of a needle through the scrotal skin into the testicle, under local anesthesia and in an outpatient setting. Technical variations have been described regarding the gauge of the needle and the number of samples taken, aiming to minimize the rates of complications and increase the success in sperm retrieval (24). The investigation of fertility potential and the presence of foci of active spermatogenesis in patients with NOA, by means of testicular aspiration biopsy, was first described in 1965. Since then, other authors have defined the use of TfNA as a routinely used diagnostic method in the quantitative and qualitative assessment of testicular morphology and presence of spermatogenesis (25).

To perform a TfNA, a cord block is performed by injecting 10 mL of local anesthesia into the spermatic cord, the region of the vas and scrotal skin where the biopsy needle will pass with a 25-gauge needle. Pull the syringe plunger to create negative pressure while moving the tip of the needle in and out of the testis; the stroke size is ~8–10 mm in an oblique plane, thereby, disrupting the seminiferous tubules and sampling different testicular areas. Maintain suction while the needle is withdrawn to remove small segments by flushing the needle with sperm wash medium. The sample is sent to the andrology lab, and a sample is assessed by Papanicolau stain (24).

Turek and colleagues conducted a prospective study of 16 patients diagnosed with azoospermia (both non-obstructive and obstructive) at a university infertility clinic in California. TfNA was compared to testicular biopsy for the ability to detect testicular sperm. The results showed that adequate samples with the use of TfNA were obtained in 91.3% of the total. TfNA showed greater sensitivity and equal specificity than testicular biopsy for sperm detection. Among men with NOA who underwent TfNA mapping, 33% had sperm “spots” detected at sites distant from those sites with negative biopsy, and in 1 case a pregnancy was achieved through a later biopsy performed and SR, directed by TfNA prior (26).

The main advantages of fine needle aspiration (FNA) and percutaneous testicular biopsy (TfNA) techniques are simplicity, low cost and less tissue damage when compared to other SR procedures. One strategy used to find sperm in patients with NOA is TfNA “mapping,” which obtains systematic tissue samples in three dimensions along the testis, using a fine needle. The cytological analysis of the material, through Papanicolaou staining, allows the identification of precursor cells (spermatogonia, primary spermatocytes and spermatids) in addition to sperm. This mapping serves as a guide for performing retrieval techniques, increasing success rates, as it directs regions with the presence of sperm to the c-TESE (26–29).

The reported success rates regarding the finding of sperm in patients with NOA after the use of testicular mapping with TfNA vary from 47 to 68%, depending on the population studied (29–31). A retrospective study conducted by Jarvis et al. aimed to assess the ability of TfNA mapping to find sperm and guide SR after micro-TESE failure in men with non-obstructive azoospermia. The authors mapped a total of 2,825 testicular sites in 82 men who failed micro-TESE. At least one site with mature sperm was found by mapping in 29.3% of men with previous micro-TESE failure. A 100% testicular retrieval rate was obtained after retrieval micro-TESE guided by TfNA mapping (32).

### Testicular sperm aspiration (TESA)

TESA is a testicular sperm aspiration procedure performed on an outpatient basis, usually with intravenous sedation. The technique consists of stabilizing the testicle in the scrotum, where a large-caliber needle (40 × 12 mm) is then introduced percutaneously into the testis, creating negative pressure to aspirate testicular tissue. The needle is usually inserted at the anteromedial or anterolateral portion of the superior testicular pole, in an oblique angle toward the medium and lower poles. Negative pressure is created by pulling the syringe plunger while the tip of the needle is moved in and out the testis in an oblique plane to disrupt the seminiferous tubules and sample different areas. The specimen is flushed into a tube containing warm sperm medium, and immediately transferred to the laboratory for microscopic examination. Simple pressure applied to the aspiration site is sufficient for hemostasis; the recovery time is ~24 h, with low rates of complications including bleeding (1%) and infection (1%) (33).

A case report presented by Lewin et al. reported for the first time the retrieval of mature sperm for treatment of a 30-year-old male with hypergonadotropic azoospermia and maturation arrest due to cryptorchidism. Multiple aspirations with 21–23 Gauge needles were performed and 8 mature sperm were captured for ICSI. After this procedure, a mature oocyte was fertilized and transferred into the uterus 48 h after its recovery, with successful delivery of a full-term child weighing 3,300g. The authors demonstrated for the first time that this approach could be effective in cases of hypergonadotrophic azoospermia with testicular failure (34).

In a Canadian study designed to compare SR outcomes by TESA or micro-TESE in men with NOA, the authors showed better SR success rates in patients undergoing micro-TESE, when compared to those submitted to TESA (88 vs. 25%, *p* = 0.0006). However, sperm concentration was significantly higher in men undergoing TESA when compared to those undergoing micro-TESE (1.2 ± 1.5 × 106/ml vs. 0.3 ± 0.5 × 106/ml, *p* = 0.012) (35).

Finally, the results of a systematic review with meta-analysis aimed at investigating the differences in SR outcomes between micro-TESE, conventional testicular sperm extraction (c-TESE) and TESA techniques in men with NOA showed that the rate of success of sperm retrieval by c-TESE was 56% (95% CI, 50–61%; *p* = 0.02) and 28% for TESA (95% CI, 19–39%; p <0.01). Therefore, in this study, the performance of c-TESE for the SR outcome was 2.0 times higher (95% CI; 1.8–2.2) compared to TESA (36).

In addition to the general decrease in sperm yield in NOA compared to OA, it has previously been demonstrated that sperm production in men with NOA can be focal, a fact that makes TESA less effective than c-TESE procedures for successful retrieval of sperm. However, even the most invasive c-TESE procedures may not find sperm if performed blindly or randomly. To address the problem of focal sperm production, different strategies have been developed for sperm localization in men with NOA, minimizing testicular damage (30, 37).

### Conventional testicular sperm extraction (c-TESE)

C-TESE for SR and fertilization *via* ICSI was first reported by Devroey and his colleagues who treated 15 patients with a diagnosis of testicular failure with the combination of c-TESE with ICSI. The authors showed that of the 182 injected oocytes, binuclear fertilization was observed in 47.8%, 57 embryos (65.5%) were obtained for transfer or cryopreservation, and the implantation rate was 18% (12).

Since then, c-TESE has been used both for the diagnosis of NOA and for sperm retrieval, and can be performed through a single or multiple incision in the tunica albuginea, without the aid of optical magnification. C-TESE can be performed under either local anesthesia with or without intravenous sedation or epidural anesthesia. Usually, a transverse 2 cm incision is made through the anterior scrotal skin, dartos and tunica vaginalis. The albuginea is incised for ~1 cm. Gentle pressure is made onto the testis to extrude testicular parenchyma. A small fragment (~5 × 5 mm) is excised with sharp scissors and placed in sperm culture media. A single or multiple specimens can be extracted from the same incision (38). Through multiple c-TESE, the tunica albuginea is incised transversely in several regions of the testicular poles, followed by a light compression and excision of the protruding tissue. The collected testicular samples are sent to the laboratory for immediate processing and analysis using microscopy, followed by closure of the tunica albuginea with non-absorbable suture (12).

The appropriate number of biopsies taken with c-TESE remains controversial among studies; however the single-incision procedure has been refuted by many authors, who claim that the presence of regions with minimal spermatogenesis foci may go unnoticed with the use of this technique. Furthermore, the number of biopsies required for sperm retrieval is significantly higher in cases of arrest of maturation and SCO compared to patients with hypospermatogenesis (37, 39).

In a prospective study conducted in Egypt the authors compared 216 patients undergoing bilateral single-incision testicular biopsy (for histopathological analysis and sperm retrieval) with 100 patients undergoing multiple sampling with a maximum of 4 samples per testis. This study showed an SR rate of 37.5 and 49% among the group of patients undergoing single and multiple biopsy, respectively. Furthermore, c-TESE with multiple sampling showed a significantly higher retrieval rate in all histopathological groups, except for SCO, tubular sclerosis and Klinefelter pattern (40).

Postoperative complication rates vary according to the SR technique applied, with an incidence ranging from 0 to 70%, including persistent pain, edema, infection, hydrocele, hematoma, androgen deficiency to atrophy and testicular failure (41–44). Intratesticular hematoma has been reported in most patients undergoing c-TESE with single or multiple incisions, based on ultrasound assessment performed after the procedure. However, most cases resolve spontaneously, without significantly compromising testicular function (43). However, some authors have reported that excision of a large volume of testicular parenchyma by c-TESE is associated with a high risk of transient or permanent reduction in testosterone levels due to testicular devascularization (17, 42).

Recent studies assessing patients with NOA have found an overall success rate in SR, ranging from 30 to 50%, with testicular sperm obtaining in all etiologic categories, encompassing cryptorchidism, orchitis, genetics, radiotherapy or chemotherapy, and idiopathic (9, 13, 18, 41, 45).

In addition to the low rates of complications associated with the procedure, c-TESE is the most used SR technique in patients with NOA. In a systematic review conducted by Donoso et al., where 24 observational studies were included, assessing c-TESE results in patients with NOA, the authors reported a mean success rate measured by sample size of ~49.5% (95% CI, 49.0–49.9) (18).

### Microdissection testicular sperm extraction (micro-TESE)

The technique called micro-TESE was initially described by Schlegel in 1999. This technique emerged with the proposal to be the gold standard for SR, because it is minimally invasive, safe and limits the impairment of testicular function, with a high sperm retrieval rate. With the use of a surgical microscope, during testicular exploration, the vascular supply of the testes is identified and preserved. The seminiferous tubules most likely to contain sperm are identified and selected through differences in size and opacity, for extraction and SR (45).

In the first report on the use of this technique, Schlegel et al. compared 22 patients undergoing c-TESE with multiple samples with a group of 27 men undergoing the micro-TESE approach. The authors demonstrated a significant increase in the SR rate when micro-TESE was used (63 vs. 45%, *p* < 0.05). No information related to the histological pattern found in the two groups was mentioned. However, less testicular tissue was extracted with the micro-TESE approach (9.4 vs. 720 mg, *p* < 0.01). This suggests that micro-TESE may improve sperm retrieval in men with NOA when comparing the results with other previously described biopsy techniques (46).

A prospective comparative study carried out by Maglia et al. in Italy analyzed 49 European-Caucasian men diagnosed with NOA undergoing micro-TESE and 96 undergoing conventional TESE. The authors compared the retrieval rate between micro-TESE and c-TESE and sought to identify candidates who could benefit from micro-TESE. Results showed that patients undergoing micro-TESE had significantly higher FSH levels (*p* = 0.004) and retrieval rates were similar between micro-TESE and c-TESE (49.0 vs. 41.7%, *p* = 0.40). Patients with a histopathological diagnosis of SCO syndrome, age > 35 years and FSH levels >18 mIU/mL undergoing micro-TESE had significantly higher retrieval rates (*p* = 0.0038) when compared to c-TESE (47).

In a systematic review with a recent meta-analysis, which assessed 21,404 patients in a total of 117 studies, the authors showed that c-TESE/micro-TESE in individuals with NOA resulted in an SR rate of up to 50%, with no differences when c-TESE was compared to micro-TESE. Retrieved sperm resulted in a live birth rate of up to 28%. Although no difference between the techniques was found in the presented review, the authors point to the need for a sufficiently powerful and well-designed randomized controlled trial to compare micro-TESE with c-TESE in men with NOA (48).

However, a letter to the editor published by Esteves et al. (49) observed that the review presented by Corona et al. presents substantial evidence of bias: (1) the significantly higher percentage of patients with unfavorable prognosis submitted to micro-TESE compared to those submitted to c-TESE; (2) authors should have also looked at the prevalence of SCO patients among included studies; (3) the diagnosis of NOA relies on a diagnosis of impaired sperm production that is most reliably made with histopathology data, and; (4) the heterogeneity of published studies included in Corona's review. Despite the biases demonstrated by Esteves in the study conducted by Corona et al., both authors agree that randomized and well-designed clinical trials are needed to compare the superiority of micro-TESE over c-TESE.

The first randomized clinical trial (RCT) on surgical sperm retrieval in men with NOA comparing multiple needle-pass TESA with micro-TESE was performed between June 2017 and April 2021, with inclusion of 100 men with NOA from four centers in Denmark and Sweden. This trial compared micro-TESE and multiple needle-pass TESA in men with NOA. Spermatozoa were retrieved in 21/49 (43%) men after micro-TESE and in 11/51 (22%) men after multiple needle-pass TESA (*p* = 0,02). The combined SRR for multiple needle-pass TESA + salvage micro-TESE was 15/51 (29%), and this was not statistically significantly different from that for micro-TESE (*p* = 0,21). The overall SRR was 36/100 (36%). No complications occurred after multiple needle-pass TESA only, while 5/89 (6%) men having micro-TESE experienced a complication, including three cases of surgically drained abscesses, one case of a surgically drained scrotal hematoma, and one case with closure of a small defect in the tunica albuginea (50).

Some studies have reported that through the micro-TESE approach, it is possible to obtain a reduction in the rates of short- and long-term complications, when compared to c-TESE, related to the endocrine and exocrine function of the testes (51, 52). In particular, a lower rate of testicular hematoma and fibrosis, a lower reduction in testicular volume (>2 ml) and a lower drop in serum testosterone levels have been observed, when compared c-TESE with micro-TESE, in favor of the latter (53).

### Open testicular mapping (OTEM)

The SR technique described by Vieira et al. (15), called Open Testicular Mapping (OTEM), was inspired by Turek's technique (26) in combination with c-TESE. This minimally invasive, low-cost, and easily reproducible approach has the advantage of not requiring the use of a surgical microscope, requiring less operative time. In addition, its main features are the best amount of tissue for analysis, as provided by c-TESE, in combination with the least testicular damage, such as testicular mapping with a fine needle, without the need for an additional procedure on the day of the procedure for ICSI.

The authors of this study first described OTEM and assessed sperm retrieval rates in men with NOA. OTEM is usually performed first on the right testis, upper portion, from medial to lateral, except when the right testicle is absent. In this technique, the testicle is accessed through a median scrotal incision and multiple testicular punctures are made in the tunica albuginea using a 19-G needle. The needle is used to open a tiny hole in the tunica without syringe aspiration, just deep enough to allow, with testicular compression, protrusion of seminiferous tubules that are pulled out with the help of two microsurgical tweezers. The amount of tissue is not weighted or measured. The testicular samples are placed on a sterile Petri dish containing 0.4 ml of culture medium, minced with microsurgical scissors and analyzed under an inverted microscope using 400× magnification for the presence of spermatozoa after each collection; if enough spermatozoa to inject all retrieved oocytes are identified, the procedure is ended. If no spermatozoa are found, a new hole is made and the testicular tissue sampling is repeated. Up to six holes are made in the upper, middle and lower portions of the testes when necessary. One sample is retrieved *via* a 5-mm incision in the tunica albuginea from the middle portion and send for histological examination. If no spermatozoa are found in the first testis, the contralateral testicle is approached the same way. This study demonstrated that using an approach with multiple small biopsies, it was possible to retrieve sperm in 54% of patients in the study with NOA, in addition to demonstrating that FSH levels and testicular volume were not prognostic factors for testicular sperm retrieval (15).

Lopes et al. developed a retrospective study, using OTEM, in which 118 patients with NOA were assessed. In this study, the authors concluded that the use of a less invasive, cheaper and more accessible technique enabled a sperm retrieval rate of 55.83%, a fertilization rate of 62.11%, a clinical pregnancy rate of 46.27%, abortion rate of 6.56% and live birth rate of 44.26%, similar to those found with micro-TESE (54).

Although the results presented so far demonstrate the efficacy and effectiveness of OTEM, other well-designed, randomized controlled clinical trials are needed comparing it with the current gold standard (micro-TESE) including outcomes such as sperm retrieval rate, fertilization rates, clinical pregnancy rate, abortion rate, live birth rate, and procedure-related complications.

Table 1 summarizes the main advantages and disadvantages of each surgical method presented in this review.

**Table 1:** Advantages and disadvantages of each surgical method.

**Surgical method**	**Advantages**	**Disadvantages**
TfNA	Simplicity, low cost and less tissue damage.	Need for a second procedure for sperm recovery. Despite low rates of complications, patients may present with hemospermia, hematocele, and prolonged testicular pain.
TESA	Recovery time is ~24 h, with low rates of complications including bleeding (1%) and infection (1%).	Lower SR success rates when compared to patients submitted to micro-TESE.
c-TESE	No microsurgical expertise required. Fast and repeatable.	High risk of transient or permanent reduction in testosterone levels due to testicular devascularization. The main symptoms include persistent pain, edema, infection, hydrocele, hematoma, androgen deficiency to atrophy and testicular insufficiency and intratesticular hematoma.
micro-TESE	Higher success rates in NOA. Larger number of sperm retrieved. Low risk of complications	Surgical exploration required. Increased cost and time demanding. Microsurgical instruments and expertise required. Postoperative discomfort.
OTEM	Minimally invasive, low-cost, and easily reproducible approach not requiring the use of a surgical microscope, requiring less operative time.	Recently described technique with only one publication in a scientific journal and a presentation in congress. Description of few cases.

## Conclusions

This study reviewed the different SR techniques and their success rates, as well as the advantages and limitations of these methods.

The advantages of TESA are low cost and low invasiveness; however, as a disadvantage, it has a significantly lower SR rate compared to c-TESE and micro-TESE, considered the gold standard.

C-TESE has been used for both the diagnosis of NOA and SR, resulting in the extraction of a large amount of parenchyma and an incidence of postoperative complications ranging from 0 to 70%, including persistent pain, edema, infection, hydrocele, hematoma, androgen deficiency, and even testicular atrophy and testicular failure. Currently, c-TESE is the most used SR technique in patients with NOA, with stable SR rates in different studies, ranging from 30 to 50%.

TfNA is a low-invasive SR method, with the main advantages of simplicity, low cost and preservation of the testicular parenchyma, while sparing seminiferous tubules. Testicular mapping with TfNA is a strategy used to find sperm in patients with NOA, targeting regions with the presence of sperm to c-TESE/micro-TESE, but with reported success rates ranging from 47% to 68% and the need for two procedures.

The technique called micro-TESE has currently been reported as the gold-standard for SR, with the proposal to be less invasive, safe, with low impairment of testicular function and retrieval of around 63%. However, its superiority in relation to c-TESE remains controversial in the literature among several authors, since there is a great divergence between studies regarding retrieval rates. Furthermore, the technique requires extensive dissection of the testicular parenchyma in the search for areas with dilated and opaque seminiferous tubules. Another disadvantage is its high cost and longer operative time, due to the need of a surgical microscope and surgeons skilled in microsurgical techniques.

OTEM allows mapping and retrieving sperm in the same procedure without the need for parenchyma dissection or use of a surgical microscope, thus implies less tissue damage, lower cost and shorter operative time. The most recent studies show SR rates between 54 and 62%.

## Author contributions

MV and MB: conceptualization. MV, MB, and ES-S: project management, methodology, writing–original preparation, writing–review and editing, supervision, validation, and visualization. MB and ES-S: data collection. MB: resource management and investigation. All authors contributed to the article and approved the submitted version.

## Conflict of interest

The authors declare that the research was conducted in the absence of any commercial or financial relationships that could be construed as a potential conflict of interest.

## Publisher's note

All claims expressed in this article are solely those of the authors and do not necessarily represent those of their affiliated organizations, or those of the publisher, the editors and the reviewers. Any product that may be evaluated in this article, or claim that may be made by its manufacturer, is not guaranteed or endorsed by the publisher.
